# Wernekink Commissure Syndrome Secondary to Bilateral Caudal Paramedian Midbrain Infarction Presenting with a Unique “Heart or V” Appearance Sign: Case Report and Review of the Literature

**DOI:** 10.3389/fneur.2017.00376

**Published:** 2017-08-03

**Authors:** Chenguang Zhou, Yuanhong He, Zhiwen Chao, Yinghui Zhu, Peng Wang, Xingping Wang, Shanshan Liu, Wei Han, Jianping Wang

**Affiliations:** ^1^Department of Neurology, The Fifth Affiliated Hospital of Zhengzhou University, Zhengzhou, China

**Keywords:** Wernekink commissure syndrome, caudal paramedian midbrain infarction, hypertrophic olivary degeneration, “heart-shaped” sign, bilateral internuclear ophthalmolegia, Palatal myoclonus

## Abstract

Wernekink commissure syndrome secondary to caudal paramedian midbrain infarction (CPMI) is a rare midbrain syndrome involving the decussation of the superior cerebellar peduncle in the caudal paramedian midbrain tegmentum. The central characteristics are constant bilateral cerebellar dysfunction, variable eye movement disorders, and rare delayed palatal myoclonus. Following is a description of the case of a 60-year-old man who presented with dizziness, slurred speech, and difficulty walking. Neurological examination revealed bilateral cerebellar dysfunction and bilateral internuclear ophthalmoplegia (bilateral INO). Serial magnetic resonance imaging (MRI) revealed a lesion in the caudal paramedian midbrain with a “heart-shaped” sign on fluid-attenuation inversion recovery images and a “V-shaped” appearance on diffusion-weighted imaging (DWI). An acute CPMI with a “heart or V” appearance sign was diagnosed. Upon follow-up evaluation 3 months later, a palatal tremor accompanied by involuntary head tremor was discovered. Hypertrophy and increased signal of the bilateral inferior olivary nucleus, compatible with hypertropic olivary degeneration (HOD) were revealed during a subsequent MRI study.

## Introduction

First described by Lhermitte in 1958, Wernekink commissure syndrome presents as characteristics of bilateral cerebellar dysfunction, eye movement disorders, and palatal myoclonus (1). Although extremely rare, caudal paramedian midbrain infarction (CPMI) is a cerebrovascular event reported earlier to be associated with the Wernekink commissure syndrome (2–10). The neurological deficits resulting from CPMI may be caused by the destruction of the decussation of dentatorubrothalamic pathway as well as the dentatorubroolivary system in the midbrain. From our experience and knowledge, “heart appearance” on magnetic resonance imaging (MRI) is a unique demonstration of bilateral medial medullary infarction (11). However, “heart or V-shaped” infarction of the midbrain has not frequently been discussed in literature. In our study, a unique MRI finding of a bilateral CPMI that stimulated a “heart or V” appearance sign is reported. In addition, in our study, the clinical manifestations and the image features of 14 patients with CPMI, confirmed by MRI, were reviewed and investigated retrospectively.

## Case Report

**Figure 1:**
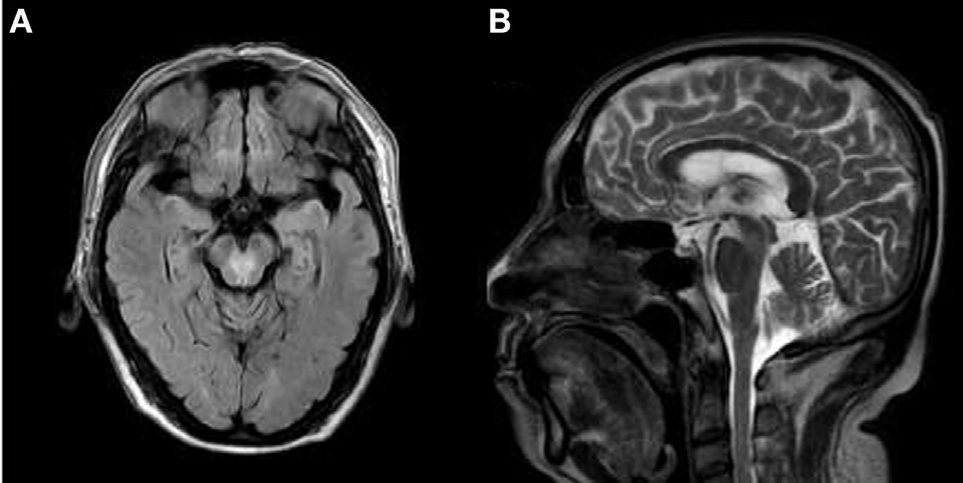
The axial brain fluid-attenuation inversion recovery (FLAIR) images **(A)** and sagittal T2-weighted images **(B)**, demonstrating a “heart-shaped” appearance area of hyperintensity located in the tegmentum of the caudal midbrain.

**Figure 2:**
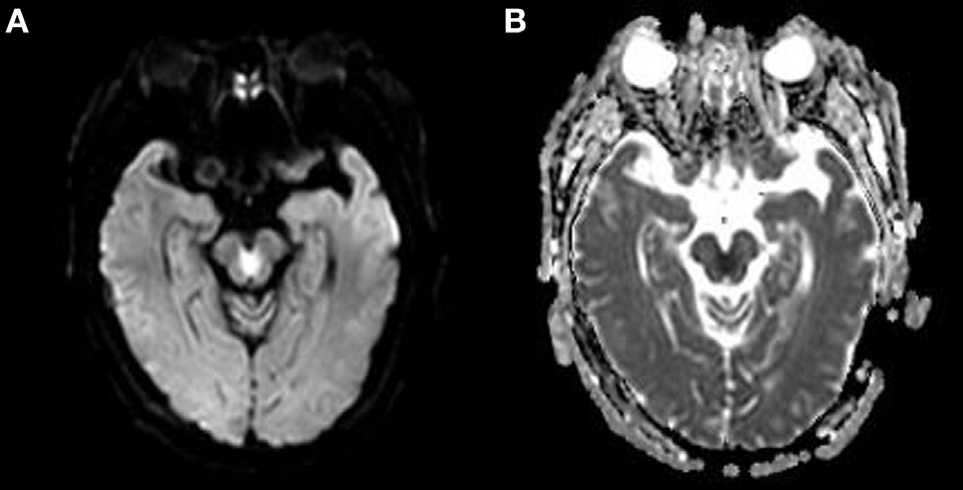
**(A)** The brain diffusion-weighted image (DWI) of the patient is shown. Note the “heart or V”-shaped lesion showing increased intensity in the tegmentum of the caudal midbrain. **(B)** The brain apparent diffusion coefficient map MRI (ADC) of the patient is shown. The “heart or V”-shaped lesion shows low intensity on ADC, consistent with acute infarction.

A 60-year-old right-handed male with a greater than 10-year history of untreated hypertension was admitted to the emergency room for sudden onset dizziness, double vision, slurred speech, and difficulty in walking for 7 h. He reported a sudden onset of dizziness and double vision during the nighttime from which he reports that he recovered within 20 min. Upon awakening the following morning, his symptoms returned and had worsened. In addition, he suffered extreme difficulty with trunk imbalance when he attempted to sit and was unable to stand without assistance due to severe disequilibrium. Upon admission, his vital signs were within normal limits except for an elevated blood pressure of 180/100 mmHg. While showing alertness and correct orientation, his speech was markedly dysarthric. Neurological examination revealed bilateral anterior internuclear ophthalmoplegia (bilateral INO) and dissociated nystagmus on horizontal gaze, in addition to bilateral upbeat nystagmus on upgaze. Convergence was impaired, although light reflexes were normal. There were no sensory deficits and his limb power was full. Finger-to-nose and heel-to-shin tests on both sides detected marked motor incoordinations and severe dysmetria. His gait was impossible to evaluate due to severe trunk ataxia. Babinski reflex was flexor response. The NIHSS score was 4. Performed 7 h after the onset of symptoms, urgent computed tomography (CT) showed lacunar infarction in bilateral basal ganglia. Initial treatment consisted of aspirin 300 mg and atorvastatin 40 mg. Laboratory examinations were within normal limits except for a uric acid level of 559 μmol/L. He was admitted to the hospital’s neurological ward for additional evaluation. A 3.0 MRI of the brain, performed 24 h after admission, showed a “heart or V”-shaped lesion in the tegmentum location of the caudal midbrain (Figure 1). Additionally, there was increased signal intensity on diffusion-weighted image MRI (Figure 2A) and low apparent diffusion coefficient map MRI (Figure 2B), in line with acute infarction. Basilar and vertebral arteries were competent in MR subtraction angiography, and irregularly narrowed bilateral posterior cerebral arteries were evident. Doppler ultrasound revealed bilateral carotid atherosclerotic plaque formation. Transthoracic echocardiography revealed left-ventricular hypertrophy, this is in accordance with his long-standing hypertension. However, 24-h Holter monitoring revealed no significant arrhythmias. These studies confirmed a diagnosis of acute ischemic stroke, and the conclusion was reached that penetrating branch vessel occlusion was the cause for the infarction. Thus, we decided to treat the patient with aspirin 100 mg and clopidogrel 75 mg, as well as atorvastatin 20 mg daily.

**Figure 3:**
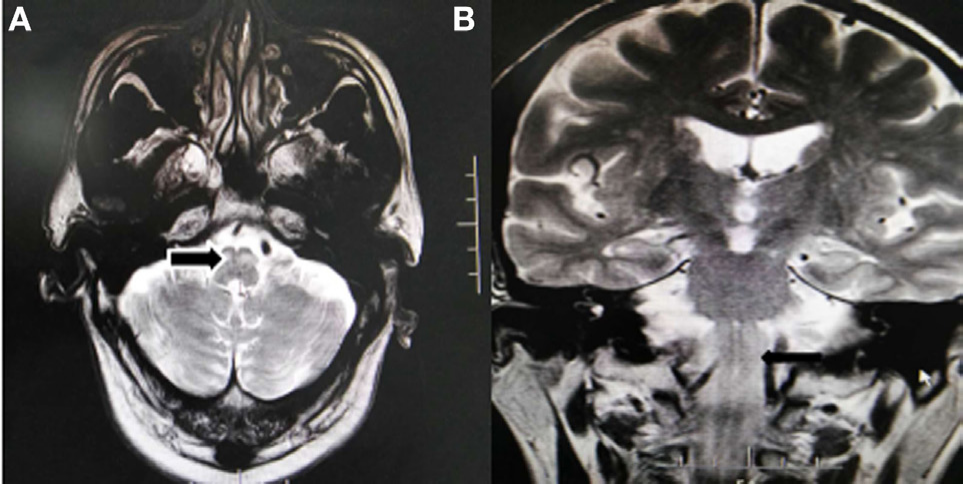
1.5T Brain MRI, axial T2-weighted images **(A)** and coronal T2-weighted images **(B)** shows the symmetric enlargement and increased signal intensity of both inferior olives (arrow).

The patient was quite sleepy during the following day despite being aggressive during the night. His aggressive behavior improved 3 days following admission. Over a 3-week period, there was a slight improvement of limb coordination and gait, and the patient was able to walk slowly with the assistance of a trolley. However, up to 3 months later, the palatal tremor accompanied with involuntary head tremor was observed. A 1.5-T MRI scanner revealed a hyperintensity and hypertrophy of the bilaterally inferior olivary nucleus on T2WI (Figure 3).

## Discussion

A small infarction in the midbrain often leads to various clinical syndromes, including Weber, Benedict, and Claude syndrome, all of which are well known to physicians. However, Wernekink commissure syndrome, secondary to CPMI, is extremely rare and commonly unrecognized. Our patient presented with classic symptoms of bilateral INO, delayed palatal tremor, and bilateral cerebellar dysfunction. Disclosed by the serial MRI was “heart or V”-shaped infarction of the caudal midbrain tegmentum, as well as bilaterally hypertropic olivary degeneration (HOD) in medulla oblongata.

**Figure 4:**
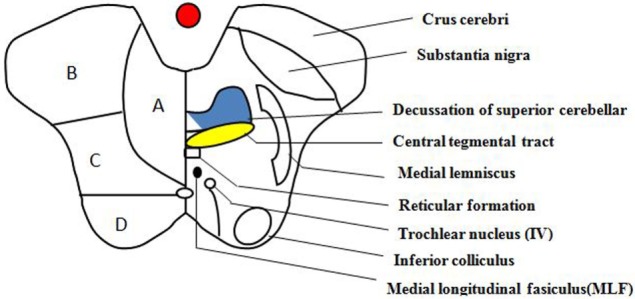
Transverse section of the lower midbrain at the level of the inferior colliculus. This schematic diagram of the midbrain depicts its arterial supply and some of the important structures within it. (A) Anteromedial, (B) anterolateral, (C) lateral, and (D) dorsal.

With a prevalence between 0.7 and 2.3% resulting from the complex supply to midbrain (12), pure midbrain infarction is very rare. While there is significant variation and overlap between various arterial territories, the midbrain is long established and classically categorized into the anteromedial (paramedian) territory, anterolateral territory, lateral territory, and dorsal arterial territory, as determined by the distribution of the penetrating artery (Figure 4) (12, 13). The paramedian area of the midbrain and thalamus are supplied through the interpeduncular fossa perforating branch, primarily arising from the tip of the basilar artery, superior cerebellar artery, as well as from the pre-communicating segment of the posterior cerebral arteries (13–15). The interpeduncular fossa perforating branches, according to the vascular supply territory, can be separated into three groups: (1) paramedian thalamic arteries (PThAs) supply irrigation to the paramedian thalami with a well-established Percheron artery variation. Occlusion of Percheron artery demonstrates a classically characteristic pattern of ischemia: bilateral paramedian thalamus accompanied or not by midbrain participation. (2) Including the medial part of the red nucleus and the third cranial nerve nuclei, superior paramedian mesencephalic arteries (SPMAs) supply the rostral midbrain. The SPMAs and PThAs are frequently originated as single or common trunk, known as the Variant IIb type of Percheron artery. (3) The common culprit vessels for infarctions in the caudal midbrain tegmentum are known as the inferior paramedian mesencephalic arteries (IPMAs), they supply the caudal paramedian midbrain tegmentum, including the Wernekink commissure, medial longitudinal fascicle (MLF), anterior portion of the periaqueductual gray matter, and reticular formation (Figure 4) (14). Consequently, occlusion of the IPMAs results in CPMI. Meanwhile, bilateral CPMI can occur when a variation exists in which bilateral IPMAs are derived from a common trunk. Additionally, the stem of the interpeduncular fossa perforating branches is subdivided into extracerebral sections and intracerebral (15). In accordance with the affected segment of perforating branches and anatomic variations, MRI, in a previous report, revealed diverse morphology of CPMI such as oval, round, oblong, and V shape lesions (Table 1). For a midbrain infarct to take on the characteristic shape of a “heart or V,” as demonstrated in our case, it is critical that the extracerebral segments of IPMAs with the variability of perforators supplying the bilateral caudal midbrain be involved. Thought to be the occlusion in the intracerebral segments or terminal segments is the oval and round lesion sparing the ventral midbrain.

**Table 1:** The summary of imaging and clinical features of Wernekink commissure syndrome secondary to CPMI.

Reference	Patient	The morphology of lesion	HOD	Ataxia (bilateral)	Eye movement disorders	Tremor and DPM
Okuda et al.^a^ (2)	57, F	Infraction (left)	No follow-up	(+)	BINO	5 weeks: tremor of the head and limbs
					Downbeat (N)	
					Impaired convergence	
Krespi et al. (3)	57, F	Round lesion (right)	(−)	(+)	RINO	No follow-up
					Upbeat (N)	
Cerrato et al. (4)	71, M	Round lesion (bilateral)	(−)	(+)	(−)	No follow-up
Liu et al. (5)						
Case 1	59, M	V-shaped lesion (bilateral)	(−)	(+)	Horizontal (N)	No follow-up
Case 2	71, F	Oblong lesion in the left	(−)	(+)	LINO	No follow-up
Sato et al. (6)	80, M	Oval lesion (bilateral)	(−)	(+)	(−)	Holems tremor
Spengos et al. (7)	67, M	V-shaped lesion (bilateral)	(−)	(+)	BINO	Holems tremor
Dai and Wasay (9)	70, M	Oval lesion (left)	(−)	(+)	LINO	Involuntary clonic type intermittent jaw opening movements
Kim et al. (10)	62, M	Oval lesion (right)	No follow-up	(+)	Upbeating (N)	No follow-up
Mossuto-Agatiello (8)						
Case 1	64, M	Oblong lesion (right)	HOD	(+)	(−)	3 years: DPM
Case 2	52, M	Oblong lesion (left)	HOD	(+)	(−)	2 months (−)
Case 3	38, M	Oval lesion (left)	HOD	(+)	Restriction of upward gaze	7 years later (−)
					Horizontal (N)	
Case 4	34, F	Oblong lesion (right)	HOD	(+)	RINO	14 months (−)
					Horizontal (N)	
Case 5	42, M	Oval lesion (right)	HOD	(+)	Bilateral third nerve paresis	5 months (−)
Present case	60, M	Heartor V-shaped lesion bilateral	HOD	(+)	BINO	3 months
					Impaired convergence	DPM
					Horizontal (N)	Tremor of the head
					Upbeating (N)	

*^a^The morphology of infarction is unclear due to old magnetic resonance imaging device*.

*HOD, hypertropic olivary degeneration; INO, internuclear ophthalmolegia; DPM, delayed palatal myoclonus; (N), nystagmus*.

Originally known as the horseshoe-shaped commissure of Wernekink, named after the German anatomist Friedrich Wernekink, was the decussation of the brachium conjunctivum. The Wernekink commissure is made up of two components, one that provides cerebrocerebellum connections *via* superior cerebellar peduncle in the midbrain was crossed ascending dentaterubrothalamic tracts. The second was crossed in the dentorubroolivary bundle that connects the dentate nucleus and interposed cerebellar nuclei with the contralateral red nucleus, located ventromedial to the brachium conjunctivum at its origin from the cerebellum. It decussates caudal to the brachium conjunctivum, followed by a dissension to the inferior olive. We are now able to clearly identify the descending bundle as Bechterews’s central tegmental tract (16). Wernekink commissure is positioned anterior to the aqueduct at the paramedian region of caudal midbrain. Adjacent structures located at the level of the inferior colliculus include the MLF, reticular formation, and trochlear nucleus (Figure 4). The clinical picture of Wernekink commissure syndrome can be divided into three categories based on the anatomical arrangement: bilateral cerebellar dysfunction, eye movement disorders, and delayed palatal myoclonus and tremor (Table 1).

All previously reported patients present with dysarthric speech, truncal ataxia, and ataxic movements of all four limbs and bilateral cerebellar dysfunction. It is very clear that bilateral cerebellar dysfunction can be attributed to decussation of the brachium conjunctivum interruption. All patients had CT- or MRI-proven critical lesion in the caudal paramedian midbrain, with the majority of them being unilateral (10/14); only 4 cases demonstrated bilateral paramedian midbrain infarction. Those patients with unilateral infarction may show bilateral cerebellar dysfunction because of interrupted dentaterubrothalamic pathways before and after the decussation. A case of midbrain infarction with acute bilateral cerebellar ataxia visualized by diffusion tensor imaging, more recently, can give a better understanding of the neurological symptoms (17).

The oculomotor signs in caudal paramedian infarction vary due to the variations in lesion shapes. Therefore, it is not surprising that INO is the most common sign of eye movement disorders in the CPMI, contributing to topical diagnosis. Six of the ten patients (the other four patients showing normal extraocular movements were excluded) had INO, with four patients documented as unilateral, and two cases, including our case, showing bilateral INO. INO results from the interruption of the internuclear pathway (MLF) connecting the nucleus of the abducens nerve with the contralateral medial rectus muscles nucleus of the oculomotor nerve. Therefore, our conclusion is that INO only appeared in line (or oblong) and V shape lesions, since MLF is located in the dorsal midbrain portion near the aqueduct. Moreover, the MLF is significant in the coordination of vertical eye movements, particular in linking vestibular input with the trochlear and oculomotor nuclei, the interstitial nucleus of Cajal, and the rostral interstitial nucleus of the MLF. Thus, the vertical nystagmus (4/14) can be exhibited on previously reported cases and our patients. In Luigi Mossuto-Agatiello’s study, five patients were included in the study and it was noted that patient 3 and 5 exhibited third nerve paresis and additional eye movement disorders. In these two cases, infarction involving caudal paramedian, there were other regions affected, including the thalamus, rostral midbrain, and cerebellum. Additionally, patient 5 suffered an injury to the third nerve after having undergone surgery for a ruptured aneurysm, making damage to the surrounding structures impossible to avoid. The authors also attribute the third nerve paresis and others to the rostral brainstem or traumatic injury to the third nerve. Therefore, the oculomotor sign could not be representative of the CPMI.

Three months later, our patients continue to show palatal myoclonus at follow-up. Secondary palatal myoclonus frequently occurs due to a lesion in the brainstem or cerebellum within the triangle of Guillain and Mollaret and has the characteristics of contractions of the levator veli palatini muscle (18). Only one patient demonstrated delayed palatal myoclonus in the earlier report. This could be related to insufficient time for follow-up in some cases. HOD, a rare condition, is characterized by a unique pattern of trans-synaptic degeneration that may or may not be accompanied by palatal myoclonus. Normally, this is caused by primary lesions in the dentorubroolivary pathway or Guillain and Mollaret’s triangle. MRI examination revealed a delayed increase in signal intensity of the bilateral inferior olives in five patients, which includes our patients and no follow-up or description in other cases. Even unilateral CPMI can result in bilateral olivary degeneration as the dentatorubroolivary fibers decussates caudal to the brachium conjunctivum. Two patients, including our patient, show hypersomnolence and sleep–wake cycle alterations, which is connected to the involvement of the reticular formation in a location dorsal to the superior cerebellar peduncles.

When a patient exhibits bilateral cerebellar dysfunction, different diagnosis, such as bilateral cerebellar infarcts, isolated bilateral middle cerebellar peduncle infarcts and acute viral cerebellitis must be taken into consideration. A sign of INO is often helpful in topical diagnosis to differentiate between CPMI and infarction in other territory. At times, the history of infection and raised cell counts of CSF examination can indicate viral cerebellitis. The bilateral cerebellar ataxia often is the sole characteristic of rostral pontine infarction (19), and the DWI of the brain must be conducted in a timely manner to make an accurate diagnosis.

## Conclusion

The Wernekink commissure syndrome, secondary to CPMI, is classically characterized by the following elements: constant bilateral cerebellar dysfunction, variable eye movement disorders, and palatal tremor or delayed HOD. An anomalous branch of IPMAs may supply both sides of the medial midbrain area thus occlusion may result in simultaneous bilateral BCPI infarcts with a heart or V appearance on MRI.

## Author Contributions

Study concept and design: CZ, YH, and JW. Acquisition, analysis, or interpretation of data: CZ, YH, JW, ZC, YZ, PW, XW, and SL. Critical revision of the manuscript for important intellectual content: CZ, YH, JW, SL, and WH. Study supervision: JW.

## Conflict of Interest Statement

We declare that the research was conducted in the absence of any commercial or financial relationships that could be construed as a potential conflict of interest. In addition, the consent was obtained from the patient for the publication of this case report.
